# Creatine Spontaneously Deposited in Oxaluria

**Published:** 1855-06

**Authors:** George W. Miltenberger

**Affiliations:** Professor of Pathological Anatomy in the University of Maryland; Baltimore


*Creatine spontaneously deposited in Oxaluria.* By GEORGE W. MILTENBERGER, Professor of Pathological Anatomy in the University of Maryland.

(Communicated in a letter to Dr. Neill.)

You will recollect that when I had the pleasure of meeting you in Philadelphia, I told you that in several cases I had lately found creatine existing in such amount or such combination in the urine, as to be deposited in fine and well-marked crystals, simply upon spontaneous evaporation. Taking into consideration the quantity of creatine which normally exists, the apparent relation between its excess, and the diseased states in which I have as yet found it, and the effects, as far as I can yet judge, of treatment, I deem it, if not of practicable importance, of sufficient pathological and physiological interest to solicit attention. I send you, by express, two slides, containing some of the crystals, so that you can judge for yourself, and if you consider it of sufficient interest, you can insert a notice or a more detailed statement of the fact in the Examiner, if you choose so to do.

In the early part of April last, I was consulted by F., a merchant engaged in and controlling a large and extensive business, for certain nervous and dyspeptic troubles, which almost entirely incapacitated him for business, and prevented the mental effort and concentration absolutely requisite in the discharge of his daily duties. In fine, he had all the symptoms which would attend a severe case of oxaluria, while, at the same time, there was marked waste and deficient nutrition, with great muscular debility. Attention being directed by the symptoms to his urine, it was found to present an unusually large deposit of oxalate of lime, (octohedra), with the oval plates or laminæ, thought by Bird to be oxalurate of lime. A drop of urine placed on a slide for examination, and covered with a thin cover-glass, was accidentally laid aside, when I was called off. Some twelve to fifteen hours afterwards I again put it under the instrument, and was struck with the unusual appearance presented. The next day, upon farther examination, when the crystalline forms were more marked, there could be no doubt, as far as microscopic appearances went, that it was creatine. It corresponded perfectly with the representations in the plates of Robin and Verdeuil, and as I have since satisfied myself with those of Funcke. I have submitted them since to the best microscopists here, all of whom agree as to their character. Attracted by the fact, I have since examined in like manner the urine in three cases of confirmed dyspepsia, all marked by more or less waste and emaciation, and muscular debility, and in the three have met with the same deposit. In another case, a boy aged 20 came into the Hospital lately, with numerous congestive abscesses in the lower limbs, a pallid, almost earthen complexion, feeble circulation, almost total inappetency, great emaciation and excessive muscular prostration. Upon careful examination of his chest, commencing tuberculosis was evident. His urine presented the richest deposit of oxalate of lime (octohedra) I have almost ever seen. Here, also, creatine was largely deposited.

All that has been necessary in these instances, has been to place the drop of urine on the slide, as for examination, and suffer it to evaporate spontaneously, when the crystals mentioned have been found in from 24 to 48 hours.

During this period I have examined various and numerous specimens of urine from other individuals, some in health, others in various morbid states, without any such result. In all the five cases where it has been seen, there have been found the crystals and oxalate of lime. If it should prove that it is not thus present, except in cases of oxaluria, and that it is not thus in excess in all cases where the oxalate is present, may it not account for the discrepant views concerning the practical import of that deposite among different observers? some believing that when the oxalate is found, it is always an indication for a particular line of treatment and a matter requiring attention; others denying that it is of any practical import.

On the other hand, it may be related to the muscular waste and debility, and its connection with the oxalate of lime be a mere coincidence, in which case, however, its physiological bearing (as to its source) would be a matter of no less interest.

As to the treatment, I have nothing new to offer. The first four were treated as cases of oxaluria, three of them with the nitro-muriatic acid; the fourth, in whom there was a predominance of the oval plates (oxalurate of lime) with nitrate of silver. In all they readily responded to the treatment, and promptly and rapidly improved, with marked diminution of the creatine.

I may be in error, but as far as my reading extends, I can find no notice of the fact that creatine has ever been found thus in urine by spontaneous evaporation. I have examined all the authorities within reach, including Lehman, and have consulted many who have been long and zealously engaged in urinary pathology, but have hitherto been unsuccessful.

*Baltimore, May 12th,* 1855.

